# Effect of climate extremes and elevation on the Tempranillo grapevine response: case study in Ribera Del Duero DO over the period 2004–2023

**DOI:** 10.1007/s00484-025-02970-z

**Published:** 2025-07-07

**Authors:** María Concepción Ramos

**Affiliations:** https://ror.org/050c3cw24 grid.15043.33 0000 0001 2163 1432 Dep. Chemistry, Physics and Environmental and Soil Sciences, University of Lleida- Agrotecnio CERCA Center, Rovira Roure 191, 25198 Lleida, Spain

**Keywords:** Acidity, Anthocyanins, Decoupling, Grape composition, Phenology, Temperature, Water availability

## Abstract

The objective of the research was to evaluate the effect of climatic extremes on the response of Tempranillo compared to the average recorded over 20 years in the Ribera del Duero DO, and to assess the main variables driving the changes and differences at different elevations. To achieve these objectives, phenological dates and grape composition were evaluated in 12 plots distributed within the DO at elevations between 725 and 915 m above sea level. Variations were related to climatic variables analysed in weather stations located in the vicinity of the plots studied. All phenological stages were advanced in the extreme hot conditions, with maturity and flowering advancing by up to 15 and 8 days, respectively. High temperatures and water deficit caused a decrease in berry weight. Grape acidity and malic acid were significantly reduced by the high temperatures during ripening (up to 8 and 30%, respectively). Phenolic composition was also affected under high temperatures, with reductions in total anthocyanins > 20%, and up to 8% in the extractable fraction, particularly at the lowest elevation, resulting in a decoupling between sugar and phenolic composition. In contrast, under extreme wet and cold conditions, acidity and total and extractable anthocyanins increased significantly (up to 18%, 20% and 2.9%, respectively). All these changes were associated with differences in maximum and minimum temperatures and water availability not only during ripening, but also during previous periods. The results allowed quantifying the effects of climatic extremes on this cultivar and give an idea of the expected effects if warmer scenarios are imposed.

**Supplementary Information:**

The online version contains supplementary material available at 10.1007/s00484-025-02970-z.

## Introduction

Grapevines are among the crops worldwide that may experience changes in the short-term future due to changes in climatic conditions, and there is widespread concern about the threat that changes in climate may have on the wine sector. One of the first aspects analysed as being influenced by changes in climate concerns the onset of phenology and the duration of phenological stages (Sadras and Petrie 2011; Palliotti et al. 2014; Fraga et al. 2016; Ruml et al. 2016; Ramos and Jones 2018; Alikadic et al. 2019; Venios et al. 2020; Bécart et al. 2022; Cayan et al. 2023; Dominguez et al. 2024). Both changes in temperature and precipitation seem to lead to an earlier phenology also affecting the length of phenological periods. An earlier harvest is expected, in most cases in warm conditions, which can also lead to changes in grape composition. A decrease in acidity has been associated with increasing temperatures (Sadras and Moran 2013; Vršič et al. 2014; Sugiura et al. 2020; De Rosas et al. 2022; Pircalabu et al. 2025), while sugar content increases due to a decrease in berry size caused by reduced water availability (Roby et al. 2004; Van Leeuwen and Destrac-Irvine 2017; de Rességuier et al. 2024; Pircalabu et al. 2025). Heat and drought affect phenolic metabolism and thus grape phenolic composition (Van Leeuwen and Destrac-Irvine 2017; Teixeira et al. 2013; Rienth et al. 2021; De Rosas et al. 2022; Ramos et al. 2024; Arrizabalaga-Arriazu et al. 2024) and the result could be a decoupling between alcoholic and phenolic maturity (Sadras and Moran 2012; Ramos and Martínez de Toda 2020; Arrizabalaga-Arriazu et al. 2024; Feifel et al. 2024). Grape composition is also affected by altitude (de Oliveira et al. 2019; Arias et al. 2022).

In addition, under warmer conditions, a decrease in yields can be expected (Yang et al. 2022; Dominguez et al. 2024). A 0.5% decrease in global vineyard area in 2023 compared to 2022 has been estimated and global wine production in 2023 suffered a 10% decrease compared to 2022 worldwide due to extreme weather conditions and fungal diseases (OIV 2023). In Spain, which is one of the countries where vines have been grown for centuries and have the largest vineyard area in the world, the area has decreased by 1% in 2023 compared to 2022 (OIV 2023). However, as each variety is adapted to specific conditions (Jones 2012), the response of each cultivar to changes in the climatic conditions may be different. In some winegrowing areas of Spain and for some varieties, yields in 2023 were not lower than in 2022 and there were other years in the series with lower values. Moreover, yields in some areas are regulated by the controlled designation of origin itself, so additional information should be analysed to assess the effects of climate change.

Some projections associated with climate change have already been made for different areas (Fraga et al. 2016; Teslić et al. 2019; Monteverde and De Sales 2020; Omazić et al. 2020; Koufos et al. 2022; Skahill et al. 2023; among others). However, although some surveys have been conducted on the effects of extreme heat for perennial crops (Parker et al. 2020), there is less information on the specific effect and consequences for vines and grape quality of extreme weather conditions, which are being recorded with increasing frequency. This research attempts to deepen this knowledge for the ‘Tempranillo’ variety. This red wine grape variety was originated from a spontaneous hybridisation between the varieties ‘Benedicto’ and Albillo Mayor (Ibañez et al. 2012) and is the fifth most cultivated variety in the world (OIV 2017). It is the dominant red cultivar in Spain (OIV 2017; Anderson and Nelgen 2020), which is the country that contributes the largest cultivated area of this variety in the world (around 89%).

Tempranillo has a short vegetative cycle, with early budding and early ripening. It is sensitive to extreme drought and can produce wines with high alcohol content and low acidity in warm climates. The Ribera del Duero DO is one of the Spanish winegrowing areas where this variety is dominant and where yields were severely affected in some years during the last decades but were not lower in 2023 than in 2022 (riberadelduero.es/consejo-regulador/estadisticas). The main objectives of this research focused on analysing the response of Tempranillo under extreme climatic conditions by (a) evaluating the response of this cultivar under extreme conditions compared to the average, (b) analysing which are the main variables driving the changes and (c) analysing if there are differences in the response at different elevations. To achieve these objectives, grape phenology and composition were evaluated during the period 2004–2023 in three zones located at different elevations in the Ribera del Duero DO. The climatic characteristics during the period and their effect on grapevine response were evaluated.

## Materials and methods

### Study area

**Fig. 1:**
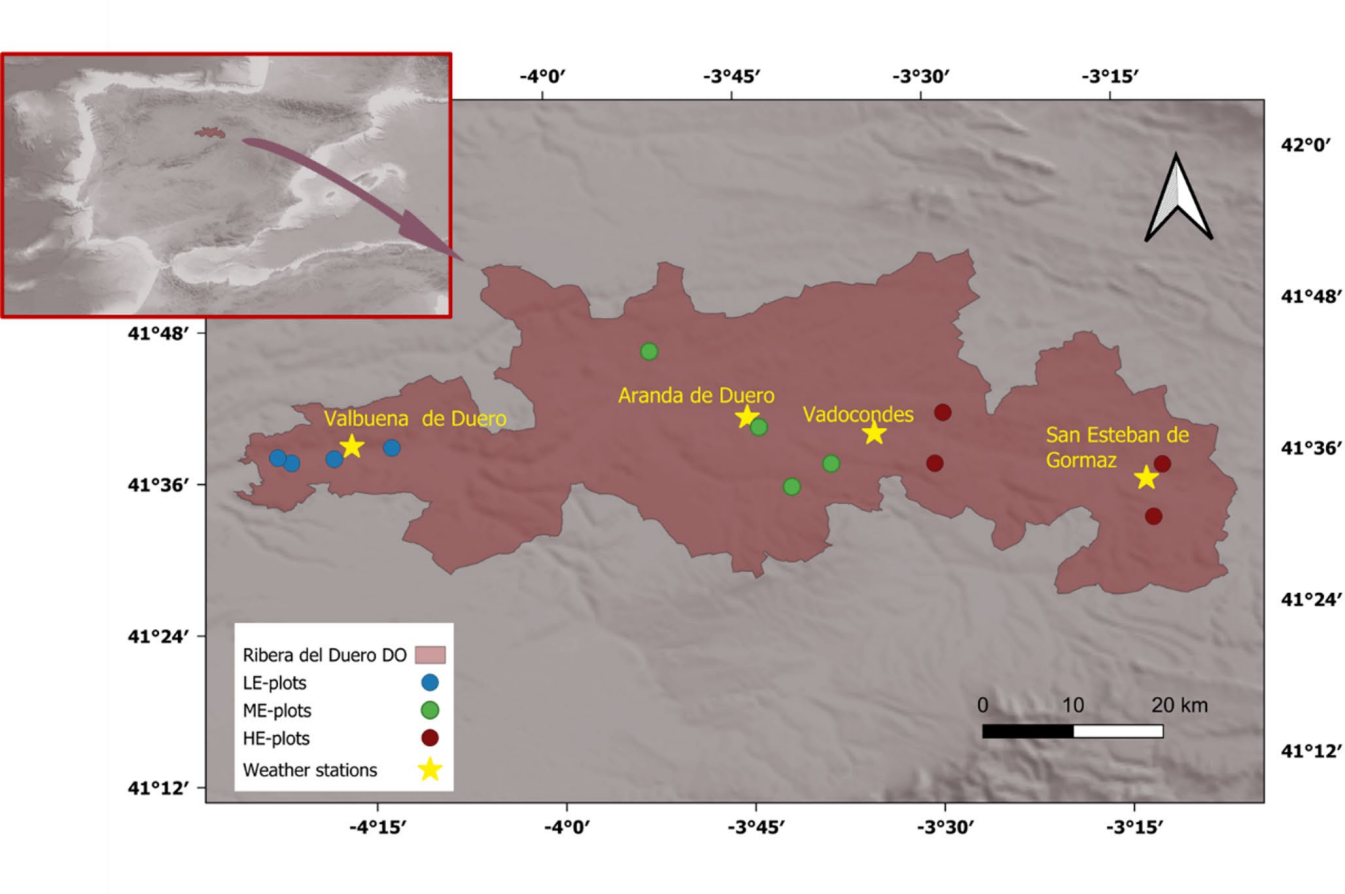
Location of the study area and plots and weather station used in this research

The study was conducted in the Ribera del Duero DO, where vines have been cultivated since Roman times. The DO covers 26,123 ha along approximately 115 km of the Duero River (Fig. 1) with elevation differences from about 700 m to more than 1000 m above sea level (a.s.l.). The geology of the area consists of a large plateau formed by a large basement filled with layers of ochre and red sandy and silty clays (Tertiary deposits) and middle and lower terraces of the river Duero (Quaternary). The main soils types according to Soil Taxonomy (USDA) are Typic Xerofluvent (in the alluvial deposits) and Typic Xerochrept, Calcixerollic Xerochrept and Calcic Haploxeralf (in the middle and lower terraces of the Duero River) (Gómez and Sotés 1992). The climate is temperate (CSb-CSa, according to the Köppen classification) with dry and warm or hot summers. The average annual temperature ranges between 10 and 12.5 °C, with average maximum temperatures between 15 °C and 20 °C and average minimum temperatures around 5.0 °C. Average annual rainfall ranges between 413 and 519 mm, with the main rainy periods in April-May and October-November-December (AEMET-IMIP 2011).

Three zones of the Ribera del Duero DO were selected, covering different elevation ranges (725–765, 830–845 and 880–915 m). For each zone, climatic information during the 2004–2023 period and vine information of different plots in each zone (Fig. 1) referred to the same period were analysed.

### Climatic information

Climatic information was recorded for each zone from four weather stations belonging to the ITACyL (Red SIAR-AEMET) located in the vicinity of the studied plots (Valbuena de Duero (394532, 4611160, elevation 740 m a.s.l.), Aranda de Duero (438164,4612943, elevation 790 m a.s.l); Vadocondes (452061, 4609855, elevation 811) and San Esteban de Gormáz (481821, 4601765, elevation 852 m a.s.l.) (Fig. 1). Climatic information included average (Tm), maximum (Tmax) and minimum (Tmin) daily temperatures and precipitation (P) as well as potential evapotranspiration estimated according to Penman Monteith. Crop evapotranspiration (ETc) was assessed for each site from potential evapotranspiration and crop coefficients proposed by Allen and Pereira (2009). The information was averaged for the growing season and for periods between phenological dates for each year and location for the period 2004–2023. In addition, cumulative degree days (GDD) and the precipitation-evapotranspiration index (P-ETc) were analysed for each period. The number of days with Tmax > 30 ⁰C and Tmax > 35 ⁰C and with Tmin < 0 ⁰C, was also analysed.

### Vine information

Tempranillo is the main variety cultivated in the Ribera del Duero DO, which cover approximately 95% of the vineyards. Other varieties authorised in the DO are Garnacha tinta, Merlot, Malbec, Cabernet Sauvigon and Albillo Mayor. Vines in the area are conducted in goblet (traditional system) and in trellis, which is increasing in the last decades as it favours the mechanization of the labours. The regulation in the DO mark a minimum number of 2000 plants/ha and establishes a maximum production of 7,000 kg/ha. Most of vines are cultivated in rainfed conditions, with irrigation only authorized in situations where it is necessary to achieve the objective of quality characteristic of the area. This research refers to the main variety (Tempranillo), which was analysed in plots located in the zones at the three selected elevations. For each plot, the phenological dates of budbreak, flowering, veraison (corresponding respectively to stages C, I and M according to Baggiolini (1952)), and maturity were collected, and the composition of the grapes (sugar content expressed as probable alcoholic strength (PVAD), titratable acidity (TAc), malic acid (MAc), total and extractable anthocyanins (TAnt and EAnt), colour intensity (CI) and berry weight (BW, expressed as weight of 100 berries)) were analysed for the period 2004–2023. All parameters were analysed according to the methods recommended by the OIV (OIV 2004) (the alcoholic degree was measured by refractometry-OIV-MA-AS312-01B; TAc by titration-OIV-MA-AS313-0; MAc by colorimetry-OIV-MA-AS313-10, and the anthocyanins and the polyphenol index were quantified by measuring the absorbance at different pH at 520 nm and 280 nm, respectively). The information was supplied by the Regulatory Council of the Ribera del Duero DO. For the phenological dates, the dominant stage (> 50% of the plants in the plot) was considered for each observation. Maturity was defined as the date on which the probable alcohol content (PVAD) of around 13 ⁰B (13.8% v/v) was reached. This value was considered to make comparable the results although in some years and locations higher values were reached at harvest.

### Statistical analysis

Differences in phenological dates and grape composition between areas were evaluated by means of a Tukey test and ANOVA analysis. The years with extreme conditions were identified from the analysis of climatic indices referring to temperature and precipitation during the growing season by means of a hierarchical cluster analysis, using the Ward method and the squared Euclidean distance. The centroid analysis made it possible to classify the years of the series. The phenological dates and grape composition of the different groups of years established in the cluster analysis were assessed. The variables that contributed most to the response were assessed by factor analysis, using principal component factoring and Varimax rotation. Loading matrices were evaluated to identify relationships between grape parameters and climatic variables.

## Results

### Climate variability

The three zones analysed showed differences in the average temperature, with greater differences in the Tmin than in the Tmax. Referring to the months from April to September, when the vegetative period takes place in the area, the average Tmax for the period ranged from 25.2 to 25.6 ⁰C. The average Tmin ranged between 7.2 and 9.0 ⁰C, with greater differences between localities but with a smaller range within each of them (between 2.1 and 2.7 ºC), and with years in which the highest Tmin was up to 1.5 and 1.7 ºC higher than the average, respectively at the lowest and highest elevations. Differences between meteorological stations were greater than for Tmax (with a greater range between locations), while differences between maximum and mean values ranged between 1.5 and 1.7 ⁰C (greater differences at the highest elevation). During the 20 years analysed (2004–2023), a high variability was observed with Tmax of up to 27.2–27.3 ºC, and differences between years of up to 3.8 and 4 ⁰C and with years in which the Tmax was up to 2 ºC higher than average, with minimal differences between zones. The years with the highest Tmax were 2017, 2022 and 2023 and higher Tmin were recorded not only in these years but also in 2006, 2011, 2015, 2018 and 2020.

The number of days per year with Tmax > 30 ⁰C ranged between 22 and 72 and those with Tmax > 35 ºC between 1 and 33, reaching the highest values in 2022 (between 29 and 31 days, depending on location), followed by 2023 (13), 2017 (between 20 and 16 days), 2018 (between 9 and 16 days), 2020 (between 8 and 9 days) and 2012 (between 9 and 12 days).

In terms of precipitation, the average precipitation during the April-September period ranged from 173.7 to 207.8 mm, with slightly higher values at higher elevations, but with a large variability between years. This precipitation represented between 42 and 48% of the annual precipitation (referred to the hydrological year), whose average ranged between 424 and 434 mm, between localities, but with values of up to 574 mm and 634 mm, respectively in the weather stations located at lower and higher elevations. The years with the wettest growing season were 2007, 2008, 2013, 2018 and 2021, but in terms of the hydrological year (including the dormant period), 2010 was also wet. In contrast, the driest years were 2005, 2009, 2012, 2017 and 2022, not only during the growing season but also during the dormant period, implying low water reserves. In other years (such as 2014, 2015 and 2016), little precipitation was accumulated during the growing season, but a large amount of water accumulated during the dormant period.

**Fig. 2:**
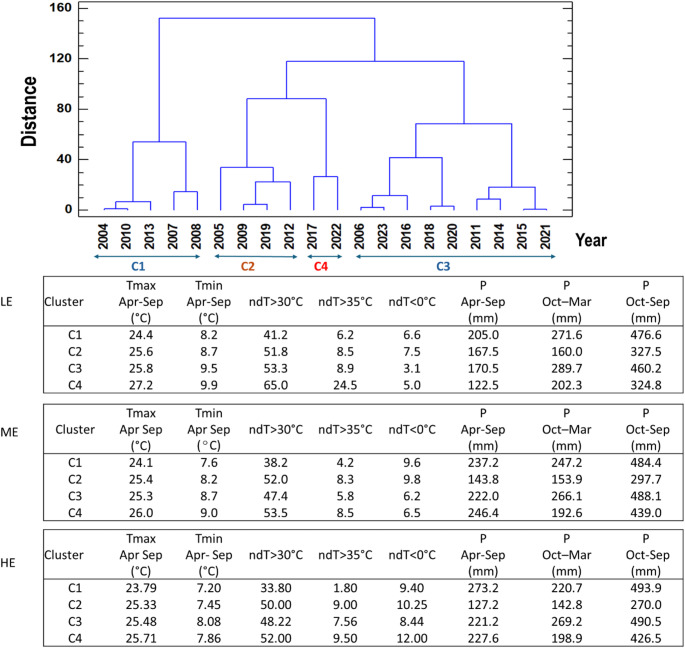
Classification of years into clusters (C1-C4) and centroids referring to maximum (Tmax) and minimum (Tmin) temperature and number of days with cold and warm extreme temperatures: T < 0 ⁰C (ndT < 0 C), T > 30 ⁰C (ndT > 30⁰C) and T > 35⁰ ⁰C (ndT > 35⁰C), and precipitation (referring to the period April-September, the hydrological year (October-September), and the dormant period (October-March)

Figure 2 shows the year classification obtained in the cluster analysis for the lower elevation (LE) weather station, west of the Ribera del Duero DO. Four clusters can be identified. On the one hand, clusters C2 and C4 grouped warm and dry years and within them, C4 grouped the warmest and driest years (years 2017 and 2022, with a high number of days with Tmax > 35 °C (25 days) and very low precipitation (< 125 mm) during the months that covered the growing season (see centroids in Fig. 2). The years included in cluster C2 were also warm and dry, both during the growing season (160 mm) and in the total year approximately (327 mm), but with fewer days with extreme temperatures. On the other hand, cluster C1 grouped the coldest years (with the lowest average Tmax and Tmin) and the wettest years (above-average rainfall throughout the year), while the years included in cluster C3 had similar average temperature characteristics to those included in cluster C2, but with above-average rainfall, particularly during the months corresponding to the dormant period (prior to the growing season). Thus, extreme conditions are included in cluster C4 (as the warmest and driest) and in cluster C1 (as the coldest and wettest). For the highest elevation weather station, the year 2022 was more directly related to the rest of the warm years, while the opposite was true for the station at intermediate elevation. The centroids of the stations at medium (ME) and high elevation (HE) are also shown in Fig. 2.

### Variability in vine phenology

**Fig. 3:**
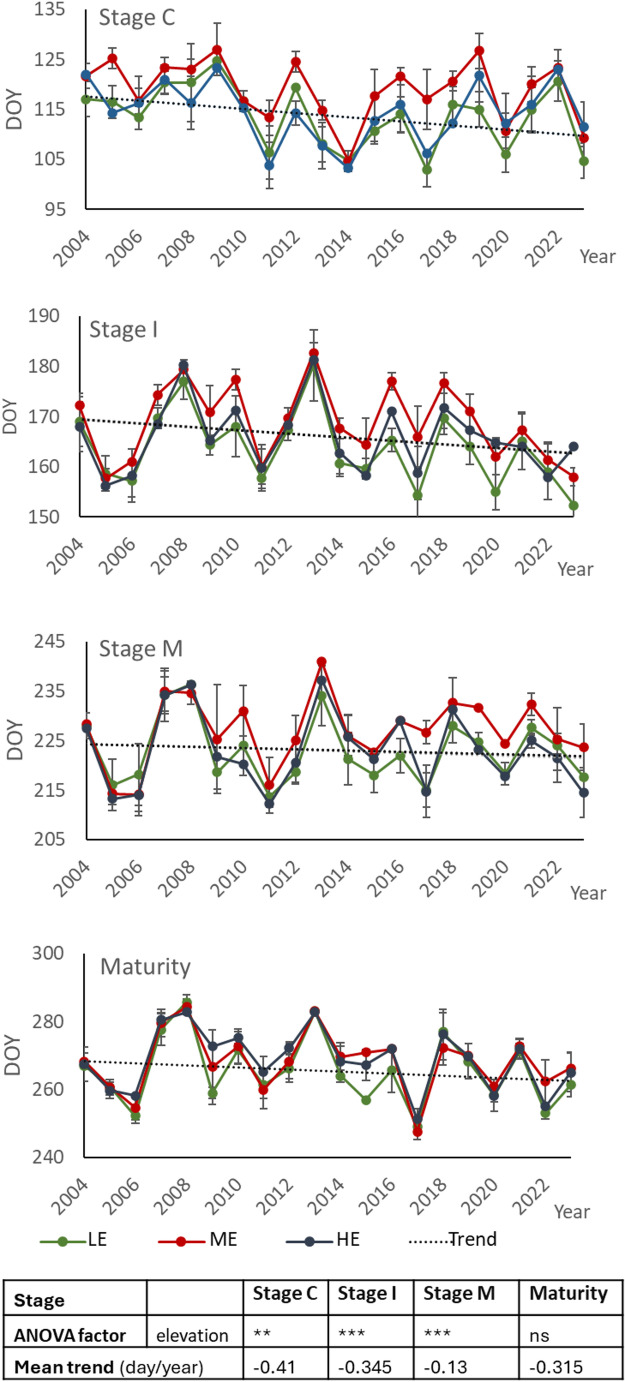
Phenologial dates of Tempranillo during the period 2004–2023 at different elevations: LE (725–765 m), ME (841–864 m) and HE (880–915 m). Data are mean ± STD. The lines represent the average trend of the dates along the period analysed. Negative sign in trend means an advance

Figure 3 shows the average phenological dates in the three zones analysed for the period 2004–2023. No significant differences in phenological dates were observed between the higher and lower elevation zones. However, later average budbreak, flowering and veraison dates were observed in the plots located at medium elevation. The earliest budbreak occurred in 2011, 2014, 2017, 2020 and 2023, while the latest budbreak occurred in 2009, in all locations. The earliest flowering occurred in 2005, 2006, 2011 and 2023 in all three locations and also in 2017, 2020 and 2022 in two of the three zones, while the latest flowering occurred in 2013 and 2008. As for veraison, the years with the earliest veraison were 2005 and 2011 in the tree zones and also in 2017 and 2023 in two of the three zones, while the latest occurred in 2008, 2007 and 2013, followed by 2018. The years with the earliest flowering and veraison were within the warm and dry year groups in all three zones. Despite the variability in the dates from year to year, an average trend of advance of all phenological stages may be observed (mean trend and changes per year are indicated in Fig. 3). Considering the average of the dates, this trend implies an advance of 8.2 days for budbreak, 6.9 days for flowering, 2.6 days for veraison and 6.3 days for maturity for the 20-year period analysed. The variations in the phenological dates with respect to the mean considering the groups of years that were defined in the cluster analysis are shown in Table 1. The changes in relation to the average are expressed in days with sign (+) when there was a delay and with sign (-) when there was an advance. Significant differences can be observed between the clusters that included the years with extreme conditions (cluster C4: very warm and dry, and cluster C1: cold and very wet).

**Table 1:** Mean phenological dates (stage C: budbreak, stage I: flowering, stage M: Veraison and maturity) for the period 2004–2023 and delay (+) or advance (-) in days for the groups of years established in the cluster analysis (clusters C1 to C4) for the three zones at different elevations (LE: low; ME: medium; HE: high)

Elevation		cluster	Stage C	Stage I	Stage M	Maturity
LE	Mean		22-Apr	11-Jun	9-Ago	21-Sep
	variation (days)	C1	+ 3 a	+ 7 a	+ 6 a	+ 10 a
		C2	+ 5 a	−1 b	−3 b	−2 b
		C3	−4 b	−3 b	−2 b	−2 b
		C4	−2 ab	−7 c	−3 b	−14 c
ME	Mean		27-Apr	16-Jun	13-Ago	24-Sep
	variation (days)	C1	+ 2 a	+ 5 a	+ 4 a	+ 8 a
		C2	+ 7 b	−2 b	−3 b	−2 b
		C3	−4 c	−3 b	−2 b	− 2 c
		C4	+ 1 a	−5 a	−4 a	−13 c
HE	Mean		23-Apr	13-Jun	10-Ago	24-Sep
	variation (days)	C1	0 a	+ 6 a	+ 5 a	+ 8 a
		C2	+ 4 b	−2 b	−3 b	−1 b
		C3	−3 c	−2 b	−2 b	−2 b
		C4	0 a	−8 c	−5 a	−15 c

*Different letters in each column indicate significant differences between clusters at 95% (Tukey test)

### Variability in grape composition

**Fig. 4:**
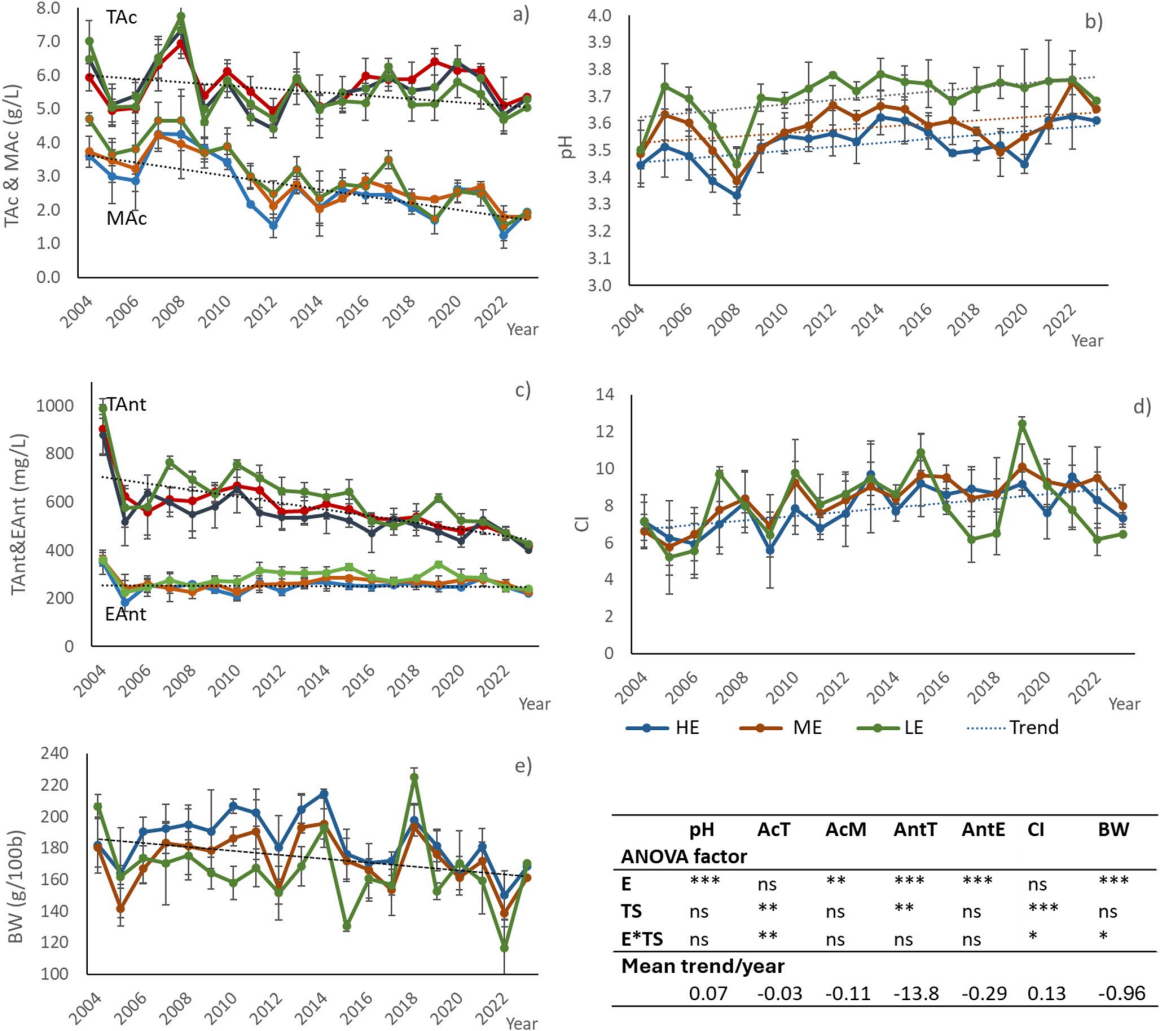
Mean and standard deviation and ANOVA (factors: elevation (E)) of grape composition (pH; TAc: titratable acidity; MAc: malic acid; TAnt total anthocyanin; EAnt: extractable anthocyanin; CI: colour intensity and BW: weight of 100 berries) in the three zones of Ribera del Duero DO at different elevations (LE: low; ME: medium and HE: high) during the period analysed (2004–2023). Lines represent the mean trend in the period. Negative sign indicates a decreasing trend

The average grape composition in each zone (average of 4 plots in each zone) recorded during the study period is shown in Fig. 4. For TAc, the highest ripening values were recorded in 2004, 2007, 2008 and 2013 and the lowest in 2005, 2006, 2012 and 2022, in the three zones. For MAc, the highest values were recorded in 2007, 2008, 2010 and the lowest in 2012 and 2022, with a decreasing trend during the period analysed and with significant differences between zones located at different elevation.

For phenolic compounds, TAnt reached the highest values in 2004, 2007 and 2010 and the lowest in 2005, 2016, 2020 and 2023. A decreasing trend was observed throughout the period analysed. There were significant differences between zones with higher values in the lower elevation. For EAnt, the lowest values were recorded in 2005, with significant differences between zones at different elevations but without a clear trend over time. For CI, the results showed greater variability between years than for EAnt, and although values were slightly higher at higher elevations, the differences between zones were not significant. The highest CI values were obtained in 2007, 2010 and 2013 and also in 2015, 2019, while the lowest values were recorded in 2005, 2006, 2009 in all three zones.

As for BW, years 2013, 2014 and 2018 presented the highest values, which corresponded with the wettest years, while the lowest values were recorded in 2005, 2012 and 2022 in all three zones, years that were very warm and dry. Berry weight was on average significantly higher at higher elevation. Despite the variability in the grape parameters from one year to another, some decreasing trends can be observed in the last decades in acidity, particularly in MAc, in TAnt and in BW, while for pH and CI the trend was to increase (mean trend lines and average changes per year are shown in Fig. 4).

The differences in the composition compared to the average considering the groups of years defined in the cluster analysis are shown in (Table 2). The results are expressed in percentage and marked with (+) when the conditions led to an increase and with (-) when resulted a decrease. Significant differences were observed between the clusters that included the extreme conditions (cluster C4: warm and dry; cluster C1: cold and wet).

**Table 2:** Mean grape composition (pH, total acidity (TAc), malic acid (MAc), total anthocyanins (TAnt), extractable anthocyanins (EAnt), colour intensity (CI) and berry weight of 100 berries (BW)) for the period 2004–2023 and decrease (-) or increase (+) in percentage related to the average in the groups of years established in the cluster analysis, for the three zones at different elevation (LE: low; ME: medium; HE: high)

			pH	TAc (g/L)	MAc (g/L)	TAnt (mg/L)	EAnt (mg/L)	CI	BW (g/100b)
LE	Mean		3.67	5.76	3.15	646	288	7.93	166.8
	variation (%)	C1	−2.9 a	18.0 b	36.8 c	27.3 d	2.9 c	11.1 c	5.3 c
		C2	0.7 b	−8.3 a	−5.3 b	−0.8 b	−0.4b	−1.2 b	−4.7 b
		C3	0.9 b	−4.7 a	−12.6 b	−9.8 c	0.4 b	−0.4 b	2.9 c
		C4	1.5 c	−7.4 a	−24.7a	−22.4 a	−8.3 a	−23.7 a	−16.8 a
ME	Mean		3.58	5.78	2.89	569	266	8.26	171.9
	variation (%)	C1	−2.0 a	11.7 b	33.8 c	19.1 c	1.8 b	2.9 b	8.1 d
		C2	0.1 b	−8.3a	−3.8 b	−3.4 b	−5.6 a	−6.9 a	−9.9 b
		C3	0.7 b	−1.9 a	−11.4 b	−5.2 b	3.0 b	2.7 b	3.7 c
		C4	1.7 c	−4.0 a	−25.7 a	−17.5 a	−6.6 a	−5.7 a	−17.3 a
HE	Mean		3.53	5.58	2.69	550	248	7.96	188.9
	variation (%)	C1	−2.1 a	13.5 c	37.1c	19.8 c	1.4 b	3.9 b	13.8 c
		C2	0.3 b	−10.9 a	−5.3 b	−3.5 b	−12.0a	−8.3a	−3.1 b
		C3	0.7 b	−1.4 b	−11.4 b	−7.2 a	4.1 b	−0.1 b	−3.2 b
		C4	1.3 c	−5.5 ab	−30.9 a	−10.2 a	1.8 b	7.5 c	−14.0 a

*Different letters in each column indicate significant differences between clusters at 95% (Tukey test)

### Relationship between grape composition and climate variables

**Fig. 5:**
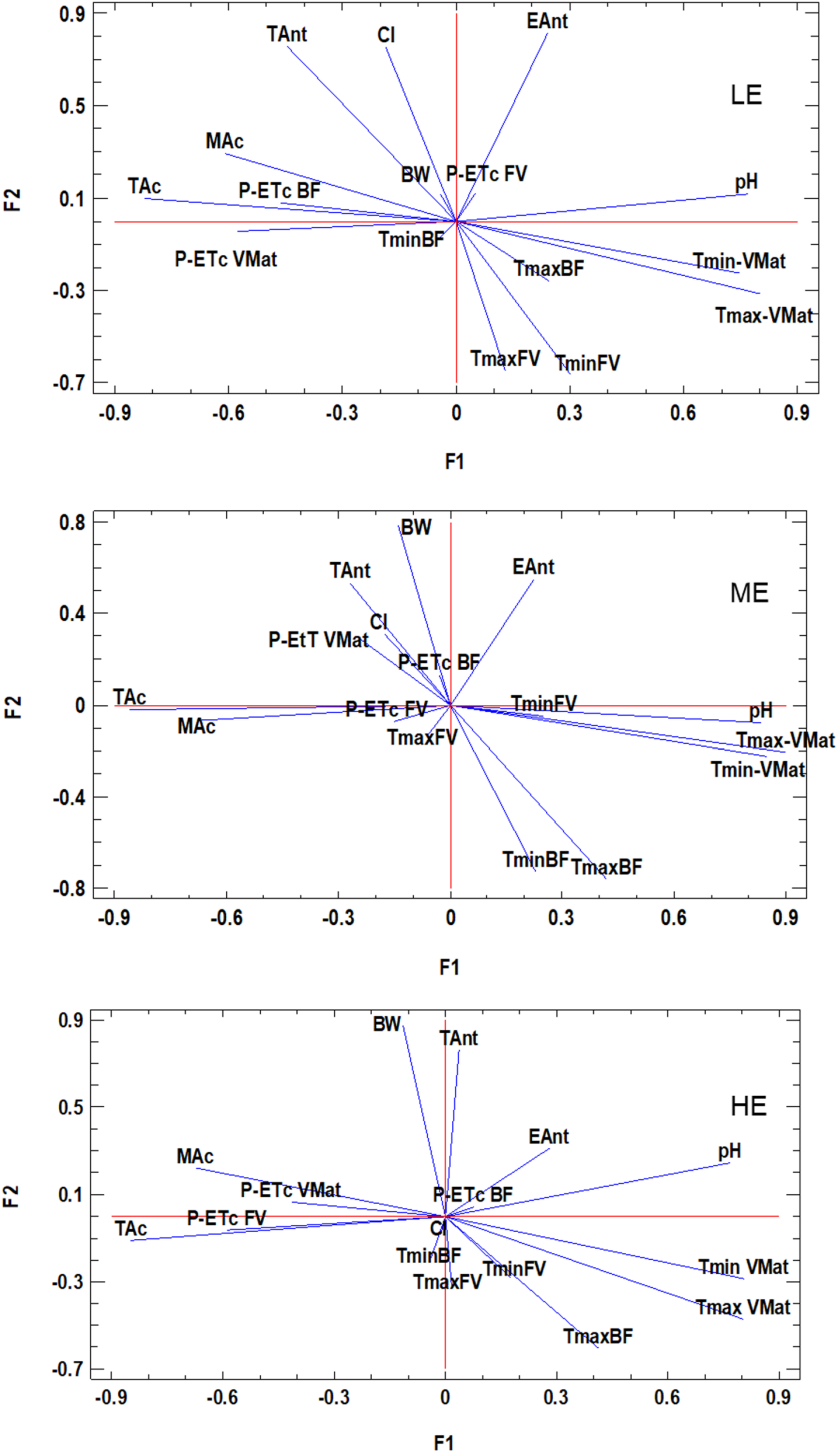
Results of factor analysis (factor 2 vs. factor 1 performed based on grape composition and climate variables recorded during the period 2004–2023 in three zones located at different elevation (LE: low; ME: medium and HE: high). (maximum temperature (Tmax), minimum temperature (Tmin); precipitation - crop evapotranspiration (P-ETc); budbreak-flowering period (BF); flowering-veraison period (FV); veraison-maturity period (VMat); pH, total acidity (TAc), malic acid (MAc), total anthocyanins (TAnt), extractable anthocyanins (EAnt), colour intensity (CI) and berry weight of 100 berries (BW))

Figure 5 and Tables SM1-3 show the results of the factor analysis carried out separately for the three zones. Five factors were retained to describe a high percentage of the variance (between 79.5 and 81.6%). Although the results were not the same in the three zones, there are several points in common. TAc and MAc are negatively affected by temperature during ripening (both Tmax and Tmin). In addition, increased ASW favours TAc. However, the periods that showed influence differed between zones (periods FV and VMat in zone HE; BF and VMat in zone LE and BF in zone ME). Opposite results were observed for pH, a variable that always appeared in the same factor. These relationships appeared in factor F1, which described between 29.1 and 38.3% of the variance. The effect of ASW during the FV period on pH was also shown in factor F5 in zone ME, a factor that described 7.4% of the variance.

As for TAnt, the results showed a negative influence of Tmax and Tmin on TAnt. However, while Tmax during the FV period was the most critical for TAnt in zones LE and ME, Tmax in the VMat period showed greater influence in zone HE. TAnt had higher loadings on factor F2, which described 14.2, 14.4 and 17.4% of the variance, respectively. EAnt was similarly negatively affected by pre-veraison temperature in zone LE, but in the other two zones it was also negatively affected by the temperature existing in previous periods (FV), as shown by the loadings on factor F3. This factor described 12.9% and 16.3% of the variance, respectively in the ME and HE zones. It is also observed that EAnt was favoured by the ASW in the FV period in zone HE. The CI index, which is also related to phenolic composition, increased with ASW in the FV period in zones ME and HE (high loadings on factor F4 and F3, respectively for zones ME and HE.

For BW, the effect of both temperature and available water was evident in the low and medium elevation zones (LE and ME), with a negative influence with increasing Tmax and ASW during the BF and FV periods. However, in zone HE, the negative effect of temperature was observed in the F2 factor, but no clear effect of ASW was observed. The highest loadings for BW appeared in factor F3 in zone LE, in factor F2 in the zone HE and in factors F3 and F5 in zone ME.

## Discussion

During the period analysed, very extreme conditions were recorded with very warm (2017 and 2022) or dry years (2005, 2009, 2012), and years in which both situations occurred together (2005, 2009, 2017, 2019 and 2022). Wet years with very low temperatures were also recorded (2004, 2007, 2008, 2013 and 2018) (Fig. 2). There were significant differences in phenology and grape composition between the two groups of years, which can be associated with differences in temperature. However, the differences were not equal for all years due to the periods with the highest extremes and the periods that most directly affected each grape parameter.

### Impacts of climatic conditions on phenology

In the warmest conditions (cluster 4), the advance of phenology was greatest for flowering (between 5 and 8 days), and for the date when maturity was reached (up to 15 days) and with the greatest advance at the highest altitude, even though harvesting took place later in this area than in those at lower elevations. In these years, Tmax throughout the growing season were very high, with average values higher than the average recorded in the 20-year period analysed, between 2.1 and 3 ⁰C higher during the BF period, between 2.3 and 2.6 ⁰C higher during the FV period and between 1.4 and 2.2 ⁰C higher during the ripening period (VMat). In addition, Tmin were also higher than the average, particularly during the FV and VMat periods (between 1.3 and 1.8 ⁰C and between 1.2 and 1.4 ⁰C, respectively). These temperatures could justify the earlier harvest in early September. Among the two years included in this cluster, 2022 was the one with the highest temperatures, with average maximum and minimum growing season temperatures (2.6 and 1.3 ⁰C, respectively, higher than in 2017). However, maturity was reached even earlier in 2017 than in 2022. This could be due to drier conditions in the VMat period (less precipitation and higher evapotranspiration). In both groups of warm years, the advancement of all phenological stages led to a shortening of the period between stages, being greater for the BF and VMat periods. For the years included in the cluster C2, the shortening was greater for the BF period, which agrees with Cameron et al. (2022). However, for the warmer years, the shortening was greatest for the VMat period.

The differences observed in the average Tmax and Tmin recorded in these two years with respect to the total average were of the same order of magnitude or even higher than the anomalies projected for 2050 under the SSP2-4.5 scenario (shared socioeconomic pathway corresponding to a balanced energy development, leading to a radiative forcing of 4.5 Wm^−2^ by 2100), estimated for the area using the ensemble of models derived from CMIP6 - Coupled Model Intercomparison Project Phase 6 for the area (Ramos 2025), and the changes in phenology were in agreement with the projected changes in phenology not only for this variety but also for other red varieties in different winegrowing areas (Alikadic et al. 2019; Ramos and Martínez de Toda 2020; Ramos et al. 2021).

In the other group, considered as warm (cluster C2), both Tmax and Tmin averages were higher than global average, but the difference ranged between 1.0 and 1.5 ⁰C during the BF periods, between 0.7 and 0.9 ⁰C during the FV period and between 0.3 and 0.8 ⁰C in the VMat period. The fact that during the earliest stage (budbreak) there was no advance, but a delay could be because the lowest temperatures recorded a higher number of days with T < 0 ⁰C just before budbreak (almost twice as many in the years included in cluster C2 than in cluster C4). Budbreak in these years of cluster C2 was even later than in the group of years considered as wet and cold (cluster C1). In these years, the average Tmax was lower than the average, particularly during the BF and VMat periods, being between 1.1 and 1.7 ⁰C lower than average in the BF period, and between 1.9 and 2.1 ⁰C lower in the VMat period. Tmin were also lower than the average, particularly during the VMat period. These values could justify the differences in the dates when flowering (5 to 7 days later), veraison (4 to 6 days) and maturity (8 to 10 days later) were reached. Among the years included in this group, 2008 and 2013 were the years with the latest veraison and maturity dates. These years had average growing season Tmax and Tmin that ranged between 24.2 and 24.7 ⁰C and between 7 and 8.6 ⁰C, respectively at the highest and lowest elevation, which average growing season Tmax and Tmin about 2.1 ⁰C and 1.4 ⁰C, respectively, below the total average. In addition, these two years, together with 2018, had the wettest growing season. The advance (or delay) in phenology recorded in these extreme conditions (hot and cold) in relation with temperature agrees with the results of other authors who related phenological dates to temperatures (Ruml et al. 2016; Fraga et al. 2016; Bock et al. 2011; Van Leeuwen and Darriet 2016; Ramos and Martínez de Toda 2020; Cameron et al. 2022). Nevertheless, as it was indicated above, temperature was not the only factor influencing phenology, but it seems that wet or dry conditions in some stages can have an additional impact.

### Impacts of climatic conditions on grape composition

As indicated by different authors, changes in phenology also influence grape composition, as earlier phenological dates shift ripening to warmer periods, altering the balance between the different processes that lead to grape ripening. Warm conditions directly affect different functions such as stomatal closure, photosynthesis or chlorophyll synthesis and degradation (Lambers et al. 2008; Venios et al. 2020) and the final grape composition (de Rosas et al. 2022; Mori et al. 2007; Sadras et al. 2013; Rienth et al. 2021; Feifel et al., 2024; de Rességuier et al. 2024). In the case of the study, differences were found in acidity and phenolic composition, as well as in berry weight under extreme conditions, when maturity was reached, which have been related to climatic variables. The warm extremes years (included in cluster C4) showed lower than average TAc values (between 4.0 and 7.4%), with the largest differences in the LE zone. However, the greatest reduction was observed in the years grouped in cluster C2, which were also warm years. These years were not as warm as those in cluster C4 but were quite dry during the growing season and also during the dormant period. In contrast, the TAc was much higher than average (between 11.7 and 18.0%) in the wettest years included in cluster C1, with larger differences at the lowest elevation.

The effect of temperature in different periods throughout the growing cycle was confirmed in the factor analysis, being the increase in Tmax and Tmin recorded during the VMat period the ones that higher decrease produced. The influence of temperature during the VMat period on TAc was similar in the three zones (Fig. 5). Li et al. (2023) indicate that temperature had no significant effect on the content of TAc in nearly ripe berries, whereas TAc content in green berries was lower at high temperatures. This could be consistent with the effect of temperature in the period prior to veraison and not during ripening. Regarding the effect of ASW, the FV period showed an impact in all three zones, while ASW in the VMat period influenced TAc in LE and HE, but in ME it was more relevant in the BF period (Fig. 5). The combination of very hot conditions, causing thermal stress, with dry conditions, causing severe water stress, may be the main reason for the high decrease in TAc, in agreement with the findings of Hewitt et al. (2023).

For MAc, the differences were much more pronounced, both in the driest and warmest years and in the wettest years. In the warmer years (cluster C4), MAc was about 25% lower than average in zones LE and ME and about 31% lower in the zone HE, while it was up to 37% higher in the wettest and colder years (years included in cluster C1) (variations ranging from 33% in ME to 36–37% in zones LE and HE). The climatic indices and the periods that had the greatest effect on MAc were the same as for TAc. MAc accumulates steadily from fruit set to veraison (Ruffner 1982a) and is favoured by relatively cool temperatures (Ruffner 1982b), so higher temperatures during that period could have a negative impact of its synthesis.

As for TAnt concentrations, in the warmer years (group C4) there was a reduction, ranging from 10.2 to 22.2%, with a greater decrease at the lower elevation and less at the higher elevation. Nevertheless, TAnt was greatest at the lowest elevation. In the other warm years (cluster C2), the differences were smaller (between 0.8 and 3.5%). However, in the years included in cluster C1 (the wettest and coldest years), TAnt was between 19.1 and 27% higher than the mean, with a greater increase at the lowest elevation. TAnt was mainly driven by Tmax and Tmin during ripening (VMat period). but also during the period prior to veraison, with a negative influence. Yamane et al. (2006) found for another variety that the period between one and three weeks after veraison was the most critical for anthocyanin accumulation when two temperatures (20 ⁰C vs. 30 ⁰C) were compared, with accumulation being higher at the lower temperature.

The results obtained in this research are in agreement with the effect of high temperatures on anthocyanin concentrations reported by de Rosas et al. (2022) for several red varieties. High temperatures (T > 30 ºC) inhibit anthocyanin synthesis and accumulation and favour its degradation (Mori et al. 2007; Yamane et al. 2006). Water status after flowering had no significant effect on TAnt, and only at ME elevation did ASW before flowering show some influence. In this regard, Uriarte et al. (2016), found similar berry phenolic composition in Tempranillo cultivated in a warm area in non-irrigated than in irrigated vines with doses that covered 25% of ETc. However, Arrizabalaga-Arriazu et al. (2024) pointed out a strong decrease on anthocyanin levels under the combination of elevated temperature, high CO_2_ levels and water deficit, although with differences between clones.

In terms of EAnt concentration, a decrease in the warmest years (cluster C4) relative to the mean was only observed in the zones LE and ME, which were the ones where the values were higher. However, in the other group of warm years (cluster C2), there was a greater decrease at the highest elevation (12%), while at the lowest elevation the variation was almost negligible (− 0.4%). In contrast, in the wettest years (cluster C1) there was an increase (between 1.4 and 2.9%). As for CI, variations followed similar trends to those of EAnt. There was also a significant reduction in the warmest years (cluster C4) at the lowest elevation (23.7%), while at the high elevation the reduction was almost negligible. In the years included in cluster C2, the opposite occurred, with the greatest reduction at the high elevation (8.3%) and the least at the lowest elevation (1.2%). In the years of cluster C1, there were higher than average values, with the variation relative to the average being greater at the lowest elevation (11.1%) than at the medium and low elevations (ranging between 6.9% and 8.3%). These two parameters did not present the same behaviour in the three zones in relation to climatic variables. Temperature during ripening showed to have an influence on EAnt in two of the three zones, with Tmin influencing more than Tmax, and with greater influence at lower elevation (LE and ME zones) than at higher elevation (HE zone). Anthocyanin extractability depends on skin cell wall breakdown (Allegro et al. 2021), which can be directly affected by temperature, and although extractability may differ between varieties, the effect of Tmin during ripening is coincident with that found in a warmer zone for Merlot (Ramos et al. 2024).

Regarding BW, in the warmest years there was a significant reduction in the zones LE and ME (of about 17%) and of about 14% in the zone HE. There was also a reduction in the warmer years included in cluster C2 (ranging from 3.1 to 9.9%). The reduction in berry weight during the years with the most extreme hot conditions led to a significant reduction in yield. Yield in 2017, was on average for Tempranillo in the Ribera de Duero DO, about 42% lower than the average in the period analysed (2004–2023) (riberadelduero.es/consejo-regulador/estadisticas). In the wettest years (cluster C1), however, mean berry weight was between 5.3 and 13.8% above average, with a large variation at higher elevations. Berry weight was clearly affected by water availability in the drier and cooler zones (ME and LE zones), but not at higher elevation (Fig. 5), although the greatest increase over the average was observed in the zone HE. Higher precipitation is usually recorded at higher altitudes, and therefore available water may show a smaller effect than in other drier zones. However, in the wet conditions recorded in the years included in cluster C1, berry weight exceeded the average and the effect was clearly higher than in the other two zones.

One of the main effects of increasing temperatures is the decoupling in the anthocyanin/sugar ratio (Martínez de Toda and Balda 2015). In the extremely warm years analysed (2017 and 2022) the TAnt/sugar ratio (expressed as PVAD) was 28.2, 26.2 and 36.7, respectively for the zones LE, ME and HEs, while on average for the 20-year periods it was 48.7, 44.2 and 42.0, respectively in the three zones. This means that, under warm conditions, this coefficient was between 12.5 and 21.4% lower than the average, with the largest differences in the zone LE and the smallest in the zone HE. In the years of cluster C2, also considered warm but not with extreme heat conditions, the ratio was close to the average, as was the case for the years included in cluster C3. On the contrary, in the very wet and cold years (cluster C1), the anthocyanin/sugar ratio was much higher than average (between 52.7 and 62.9% greater, with higher values in the LE zone.

**Fig. 6:**
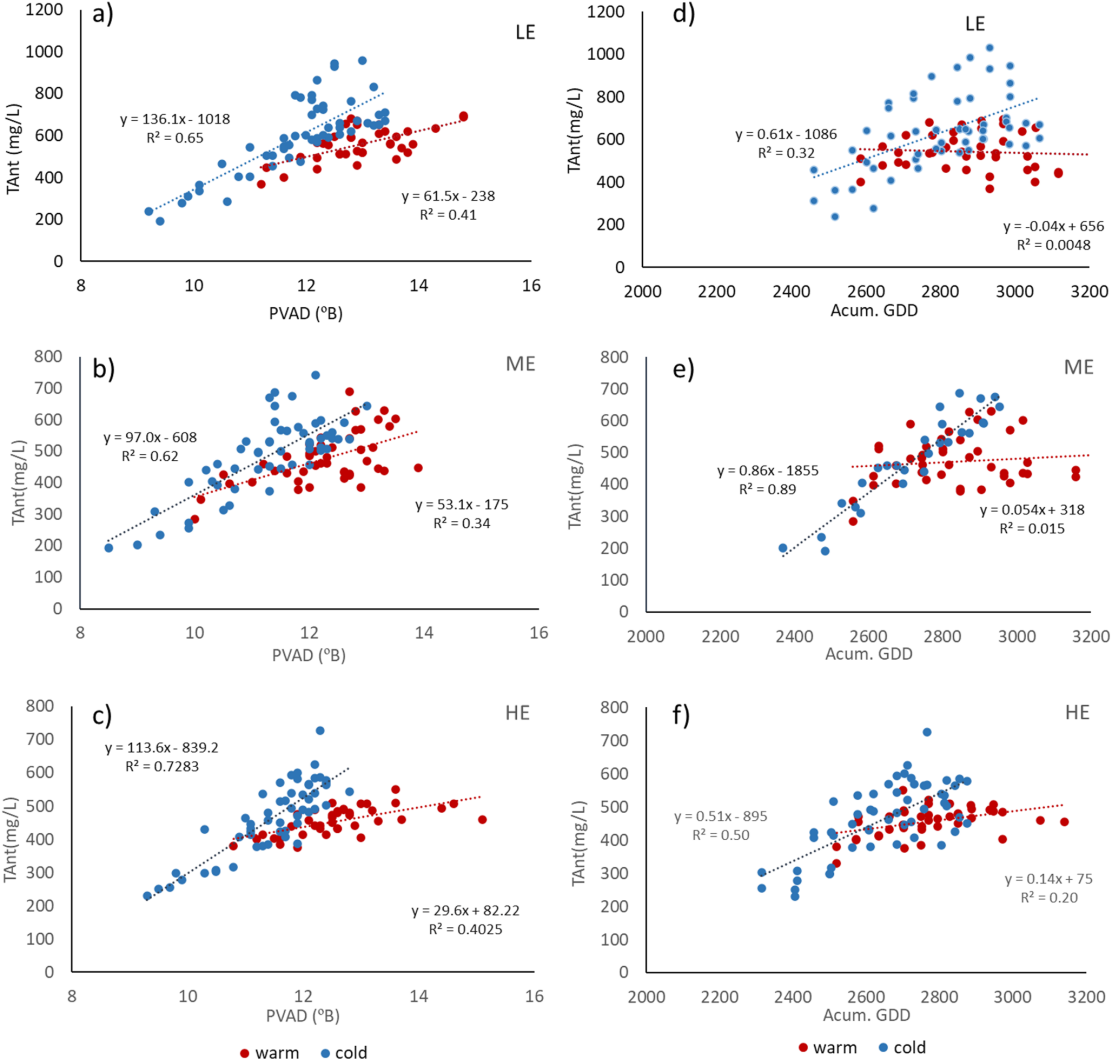
Relationship between TAnt and PVAD (**a**, **b**,**c**) and evolution of TAnt with accumulated degree days (**d**, **e**, **f**) during ripening under two different extreme conditions (warm and dry vs. cool and wet) at three elevations (LE: low; ME: medium and HE; high)

The TAnt/PVAD ratio was also driven by Tmin during the ripening period, with a decreasing relationship with increasing temperature (between 2.1 and 3.5 per 1 ⁰C increase in Tmin), implying that phenolic ripening was not adequately achieved when ripening occurred under very warm conditions. This decoupling can be observed in Fig. 6, which shows the evolution of TAnt and PVAD during ripening (from veraison to harvest), as well as the evolution of TAnt with accumulated GDD, for the warmest and coldest years, in the three zones. It can be observed that TAnt concentration did not increase at the same rate as sugar in the warmest conditions and how in those situations TAnt hardly increased with increasing cumulative temperatures or even decreased in the zone LE, where the highest TAnt concentrations were reached. Under the cold conditions, however, TAnt concentration continued increasing with cumulative degree days.

## Conclusions

The present research provides insight into the response of the Tempranillo variety in the Ribera del Duero DO under a wide range of climatic conditions and allows confirmation of the periods in which temperature and water availability have the greatest influence on grape composition. In addition, the comparison of the vines at different elevations within the area helps to increase knowledge of the potential suitability of this cultivar under different conditions. The advancement of all phenological events under the warmer and drier conditions contrasted with similar delays related to the average found under wet and dry conditions and give an idea of the potential changes in phenology that may occur under future warmer scenarios. The results indicate that the variability in grape composition is associated with differences in maximum and minimum temperatures and water availability not only during ripening, but also during earlier periods, although variation may vary depending on altitude. Grape acidity and phenolic composition as well as berry weight are negatively affected by high temperatures and low water availability, and the predicted climate change the may lead to negative effects for acidity and for the balance between sugar and phenolic composition. The results could provide useful information to winegrowers in this area, where Tempranillo is the main cultivar, to explore adaptation measures to maintain the quality and production of the grapes of this variety.

## Electronic supplementary material

Below is the link to the electronic supplementary material.

Supplementary Material 1

## Data Availability

The datasets analysed during the current study related to vines belong to Consejo Regulador Ribera del Duero (https://riberadelduero.es/viticultor/informes-de-viticultura) and the climatic data belong to ITACYL and AEMET (https://ftp.itacyl.es/Meteorologia/).
