# Supplementary material for: Effect of climate extremes and elevation on the Tempranillo grapevine response: case study in Ribera Del Duero DO over the period 2004–2023
Source: Int J Biometeorol. 2025 Jul 7;69(9):2337–52. doi: 10.1007/s00484-025-02970-z (PMC12479616; doi:10.1007/s00484-025-02970-z)
Supplement: Supplementary file 1 — Supplementary Material 1 [file 484_2025_2970_MOESM1_ESM.docx]

Table SM1. Loading matrix of obtaine after ratrion in factor performed based on grape composition and climate variables recorded during the period 2004-2023 at low elevation (LE). (maximum temperature (Tmax), minimum temperature (Tmin); precipitation - crop evapotranspiration (P-ETc); budbreak-flowering period (BF); flowering-veraison period (FV); veraison-maturity period (VMat); pH, total acidity (TAc), malic acid (MAc), total anthocyanins (TAnt), extractable anthocyanins (EAnt), colour intensity (CI) and berry weight of 100 berries (BW)).

|  | Factor1 | Factor2 | Factor3 | Factor4 | Factor5 |
| --- | --- | --- | --- | --- | --- |
| pH | **0.766** | 0.117 | 0.129 | -0.247 | 0.182 |
| TAc | **-0.820** | 0.099 | -0.287 | -0.024 | 0.083 |
| MAc | **-0.608** | 0.293 | -0.125 | 0.394 | -0.062 |
| TAnt | -0.346 | **0.757** | 0.056 | 0.314 | -0.053 |
| EAnt | 0.241 | **0.817** | -0.047 | 0.016 | 0.225 |
| CI | -0.184 | **0.752** | -0.159 | 0.027 | **0.426** |
| BW | -0.042 | 0.116 | **-0.723** | **0.533** | 0.059 |
| TmaxBF | 0.243 | -0.262 | **0.774** | 0.090 | **-0.446** |
| TminBF | -0.046 | -0.078 | 0.052 | -0.030 | **-0.847** |
| TmaxFV | 0.128 | **-0.652** | 0.537 | -0.158 | 0.176 |
| TminFV | 0.303 | **-0.668** | 0.310 | 0.008 | 0.272 |
| Tmax-VMat | **0.802** | -0.317 | 0.193 | 0.270 | -0.088 |
| Tmin-VMat | **0.746** | -0.226 | 0.439 | 0.249 | 0.067 |
| P-ETc BF | **-0.463** | 0.079 | **-0.775** | 0.047 | -0.152 |
| P-ETc FV | 0.051 | 0.120 | -0.081 | **0.898** | 0.018 |
| P-EtT VMat | **-0.577** | -0.044 | 0.124 | -0.045 | **0.429** |
| Variance (%) | 38.2 | 14.2 | 10.7 | 8.9 | 7.5 |

*Bold numbers indicate loadings >0.4.

Table SM2. Loading matrix of obtaine after ratrion in factor performed based on grape composition and climate variables recorded during the period 2004-2023 at medium elevation (ME). (maximum temperature (Tmax), minimum temperature (Tmin); precipitation - crop evapotranspiration (P-ETc); budbreak-flowering period (BF); flowering-veraison period (FV); veraison-maturity period (VMat); pH, total acidity (TAc), malic acid (MAc), total anthocyanins (TAnt), extractable anthocyanins (EAnt), colour intensity (CI) and berry weight of 100 berries (BW)).

|  | Factor1 | Factor2 | Factor3 | Factor4 | Factor5 |
| --- | --- | --- | --- | --- | --- |
| pH | **0.804** | -0.070 | -0.070 | -0.060 | **-0.421** |
| TAc | **-0.851** | 0.063 | -0.026 | 0.376 | 0.218 |
| MAc | **-0.675** | **0.577** | -0.082 | -0.366 | 0.070 |
| TAnt | -0.282 | **0.446** | **0.526** | -0.353 | 0.099 |
| EAnt | 0.222 | 0.111 | **0.542** | 0.152 | -0.102 |
| CI | -0.208 | -0.146 | 0.313 | **0.803** | -0.200 |
| BW | -0.136 | 0.270 | **0.789** | 0.083 | **0.504** |
| TmaxBF | 0.436 | -0.261 | **-0.758** | -0.096 | -0.130 |
| TminBF | 0.223 | 0.329 | **-0.729** | 0.020 | -0.028 |
| TmaxFV | -0.079 | **-0.879** | -0.124 | 0.062 | -0.301 |
| TminFV | 0.248 | **-0.913** | -0.024 | -0.003 | 0.148 |
| Tmax-VMat | **0.883** | -0.123 | -0.194 | -0.116 | 0.078 |
| Tmin-VMat | **0.854** | -0.075 | -0.214 | 0.047 | 0.192 |
| P-ETc BF | -**0.501** | **0.635** | 0.293 | 0.223 | 0.127 |
| P-ETc FV | -0.062 | 0.092 | 0.144 | 0.032 | **0.888** |
| P-EtT VMat | -0.155 | 0.206 | -0.064 | **0.817** | 0.269 |
| Variance (%) | 35.5 | 14.4 | 12.9 | 10.3 | 7.4 |

*Bold numbers indicate loadings >0.4.

Table SM3. Loading matrix of obtaine after ratrion in factor performed based on grape composition and climate variables recorded during the period 2004-2023 at high elevation (HE). (maximum temperature (Tmax), minimum temperature (Tmin); precipitation - crop evapotranspiration (P-ETc); budbreak-flowering period (BF); flowering-veraison period (FV); veraison-maturity period (VMat); pH, total acidity (TAc), malic acid (MAc), total anthocyanins (TAnt), extractable anthocyanins (EAnt), colour intensity (CI) and berry weight of 100 berries (BW)).

|  | Factor1 | Factor2 | Factor3 | Factor4 | Factor5 |
| --- | --- | --- | --- | --- | --- |
| pH | **0.768** | 0.246 | 0.203 | 0.209 | -0.099 |
| TAc | **-0.848** | -0.110 | 0.120 | -0.156 | 0.369 |
| MAc | **-0.674** | 0.220 | -0.395 | -0.187 | 0.086 |
| TAnt | 0.037 | **0.766** | -0.030 | -0.163 | 0.093 |
| EAnt | 0.279 | 0.312 | **0.691** | -0.229 | 0.182 |
| CI | -0.013 | -0.085 | **0.914** | 0.034 | 0.118 |
| BW | -0.114 | **0.878** | -0.031 | -0.183 | -0.117 |
| TmaxBF | 0.412 | **-0.606** | -0.299 | 0.177 | -0.235 |
| TminBF | -0.038 | -0.192 | **-0.482** | **-0.623** | 0.308 |
| TmaxFV | 0.017 | -0.328 | -0.053 | **0.869** | -0.028 |
| TminFV | 0.176 | -0.282 | -0.098 | **0.874** | 0.230 |
| Tmax-VMat | **0.801** | **-0.475** | -0.076 | -0.156 | 0.093 |
| Tmin-VMat | **0.804** | -0.289 | 0.030 | -0.016 | 0.143 |
| P-ETc BF | 0.075 | 0.038 | 0.063 | 0.088 | 0.926 |
| P-ETc FV | **-0.590** | -0.065 | **0.605** | 0.103 | 0.275 |
| P-ETc VMat | **-0.414** | 0.066 | 0.264 | -0.070 | **0.839** |
| Variance (%) | 29.1 | 17.4 | 16.3 | 11.3 | 7.5 |
